# Chronic Myeloid Leukemia Relapsing 25 Years after Allogenic Stem Cell Transplantation

**DOI:** 10.1155/2018/2045985

**Published:** 2018-09-23

**Authors:** Håkon Reikvam, Jørn Skavland, Stein-Erik Gullaksen, Randi Hovland, Tobias Gedde-Dahl, Øystein Bruserud, Bjørn Tore Gjertsen

**Affiliations:** ^1^Section for Hematology, Institute of Clinical Science, University of Bergen, Bergen, Norway; ^2^Section for Hematology, Department of Medicine, Haukeland University Hospital, Bergen, Norway; ^3^Department for Medical Genetics and Molecular Medicine, Haukeland University Hospital, Bergen, Norway; ^4^Section for Hematology, Institute of Clinical Medicine, University of Oslo, Oslo, Norway; ^5^Section for Hematology, Oslo University Hospital, Rikshospitalet, Oslo, Norway

## Abstract

Chronic myeloid leukemia (CML) is a myeloproliferative disorder in which neoplastic cells exhibit the Philadelphia chromosome and the related oncoprotein *BCR-ABL1*. Allogeneic stem cell transplantation (allo-SCT) was considered the first-line treatment for CML, before the introduction of tyrosine kinase inhibitors (TKIs). However, patients are at risk for relapse years after transplantation. We present a patient who relapsed 25 years after allo-SCT for chronic phase CML. Polymerase chain reaction (PCR) detected gradually evaluated levels of *BCR-ABL1* transcripts, eventually leading to the diagnosis of relapsed disease. Additional mutational analyses did not reveal mutations in the *BCR-ABL1* gene, or other cooperating mutations. The patient was successfully treated with imatinib 400 mg daily, leading to new molecular remission. The case presentation emphasizes the need for long-term follow-up of such patients and the potential benefit of initiating TKI treatment with early signs of relapse.

## 1. Introduction

Nearly 1.5 million people worldwide suffer from chronic myeloid leukemia (CML), which is morphologically characterized by an accumulation of myeloid precursor cells in the peripheral blood and bone marrow. Philadelphia chromosome-positive CML is identified by the genetic translocation t(9;22)(q34;q11.2) [1], with the fusion of the Abelson (*ABL1*) oncogene with the breakpoint cluster region (*BCR*) gene. The malignant transformation is hence caused by the acquisition of the constitutively active tyrosine kinase *BCR-ABL1* in a hematopoietic stem cell, transforming it into a leukemic stem cell (LSC) that self-renews, proliferates, and differentiates to give rise to a myeloproliferative neoplastic disease [2, 3]. CML LSCs are believed to evolve as a result of both epigenetic and genetic events and to divide less frequently, representing a reservoir for relapsed and resistant disease [2, 3].

Since the introduction of tyrosine kinase inhibitors (TKIs), targeting the kinase activity of *BCR-ABL1*, CML has transformed from a once fatal to a manageable disease for the vast majority of patients [4]. However, before the introduction of TKIs, the only potential curative treatment offering hope of long-time survival was allogenic stem-cell transplantation (allo-SCT) [5], although associated with high rates of treatment-related mortality and morbidity. Most patients who relapsed after allo-SCT would do so within the first year after transplant, although later relapses have also been described [6–9]. Here, we present a patient relapsing 25 years after initial allo-SCT of CML, emphasizing the importance of long-time follow-up for allo-transplanted patients.

## 2. Case Presentation

A previously healthy 41-year-old woman was diagnosed with CML, after presentation of symptoms caused by hypersplenism with gravity sensation under the right costal margin. Initial blood tests demonstrated severe leukocytosis 227 × 10^9^/L (normal range 4–11 × 10^9^/L) together with increased serum concentration of lactate dehydrogenase (LDH) 969 U/L (200–450 U/l) and cobalamin 2834 pmol/L (150–840 pmol/L). A blood smear demonstrated dominance of myeloid precursor with increased metamyelocytes and rods. CML was confirmed by identification of the Philadelphia chromosome t(9; 22) by using conventional G-banding analyses.

Treatment was initiated with hydroxyurea combined with interferon, and the patient reached morphological remission, then proceeded to allo-SCT for consolidation treatment. The transplantation was performed by a myeloablative condition (MAC) regime with busulphan 1 mg/kg for four days followed by cyclophosphamide 60 mg/kg for two days, followed by bone marrow-derived stem cell from her human leukocyte antigen- (HLA-) matched sister. Standard graft versus host disease (GVHD) prophylaxis by using cyclosporine A and methotrexate on day 1, 3, 6, and 11 after transplant was given [10]. No severe complications were observed after transplant, and especially, she did not develop any signs of acute or chronic GVHD.

After the development of polymerase chain reaction (PCR) analysis for *BCR-ABL1* transcripts [11], this test has been regarded as mandatory in the follow-up of CML patients [11]. Six years after the allo-SCT, an e13a2 transcript of *BCR-ABL1* was detected by nested PCR. She was therefore controlled twice yearly, without signs of progression judged from karyotyping and interphase fluorescence in situ hybridization (FISH) of 200 interphases with probes against *BCR* and *ABL1* in the bone marrow. By standardization of quantitative real-time (RT) PCR, yearly analyses were performed [11], and low but detectable transcript levels were still observed, although molecular remission (MR) levels were below MR3.

**Figure 1:**
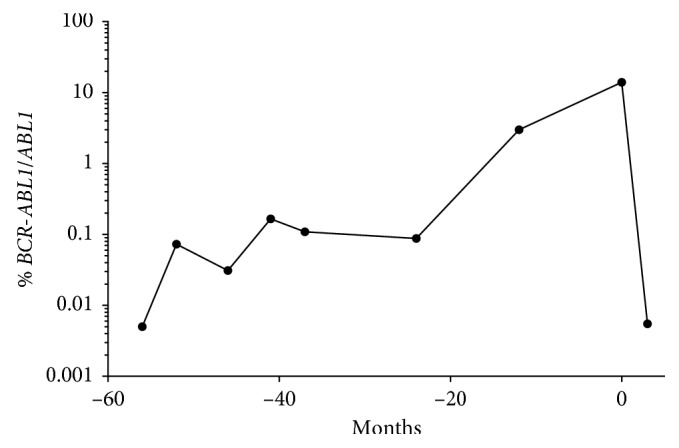
Development in *BCR-ABL1/ABL1* transcript levels in the setting of relapsed CML. The figure shows the *BCR-ABL1/ABL1* transcript levels in peripheral blood for the patient. Time point 0 represents the diagnosis of CML relapse and initiating of imatinib therapy.

Her transcript levels then suddenly increased rapidly, and she lost her MR (Figure 1). This was confirmed by analysis at two different laboratories. The patient proceeded to bone marrow examination showing normal metaphases by G-banding and only one cell with *BCR-ABL1* of 245 interphases by FISH using dual fusion probes, and this was regarded as insignificant. The bone marrow smear was hypercellular with increased myeloid precursors and megakaryocytes, although without evidence of increased myeloblasts. Hence, we maintained the diagnosis of CML with molecular relapse appearing 25 years after initial allo-SCT. The patient was screened for other mutations commonly occurring in myeloid malignancies, including mutations in *BCR-ABL1*, but no additional mutations were detected. Donor chimerism status revealed that the majority of myeloid cells (99%) were of donor origin. We thought that her relapsed disease was likely to be sensitive to treatment with first-generation TKI, and she was given imatinib 400 mg daily. She tolerated the treatment well without major side effects; after three months, she obtained MR4 status (Figure 1), and imatinib treatment was continued with regular monitoring of *BCR-ABL1* quantitative RT-PCR.

## 3. Discussion

Allo-SCT played a central role in CML treatment before the TKIs era because it was the only treatment with proven curative potential [5]. For this reason, CML was the most common indication for allo-SCT until the beginning of the new millennium. The susceptibility of CML to the graft-versus-leukemia (GVL) effect, the documented effect of donor lymphocyte infusion (DLI) in CML relapse, and the possibility to monitor minimal residual disease (MRD) were features placing this disease at the forefront of allo-SCT research. However, the introduction of imatinib, and the clearly therapeutic benefits of this treatment approach, led to a rapid decline of the transplantation rates in CML. However, several patients successfully transplanted for CML are still under follow-up worldwide. Most CML relapses after allo-SCT occurred during the first year after transplant, although late relapses, including extramedullary relapses can also be detected [6–9,12–15].

The present patient was allografted before the introduction of TKIs. She was given induction therapy with hydroxyurea and interferon, considered as the standard treatment at that time [16]. After receiving a complete morphological remission, she was allografted with an HLA-matched sibling donor. During the posttransplant follow-up, she had persistent detection of *BCR-ABL1* transfusion transcripts. The method of detecting *BCR-ABL1* transcripts has been standardized more recently [11]; hence, an accurate quantitative measurement of *BCR-ABL1* transcripts has been available only the last years before the relapse (Figure 1). However, the patient had proven detection of *BCR-ABL1* transcript for >5 years before the posttransplant relapse. The detection of such minimal residual disease (MRD) is not uncommon neither for allografted patients nor for patients treated with TKIs [17]. The detection of *BCR-ABL1* transcripts is believed to be caused by the persistence of an LSC pool in CML patients [2]. However, the clinical importance or therapeutic implications of such MRD detection is controversial, although a rapid increase in transcript levels or loosing of previous MR should wake the attention from the treating physician.

Studies have demonstrated that patients with *BCR-ABL1* expression in the hematopoietic stem cell compartment seem to have inferior survival irrespective of the disease status [18]. The quantitative RT-PCR has become widely used for monitoring minimal residual disease after allo-SCT for CML. However, most of these studies were performed using qualitative RT-PCR, and the interpretation of the results obtained has been conflicting. By the use of quantitative RT-PCR performed early within three to five years after allo-SCT, a clear relationship between *BCR-ABL1* transcript level and probability of relapse seem apparent [19].

In the 1990s, donor lymphocyte infusion (DLI) was the mainstay of treatment for posttransplant CML relapse [20, 21]. DLI induced durable responses in 60–70% of patients relapsing with chronic phase CML [21], whereas durable remissions in patients relapsing into accelerated or blast phase are less frequent [21]. The main obstacle in allografted patients is GVHD that can be a very sever complication. On the other hand, GVHD seems to be highly correlated to response rates [21, 22]. TKIs are now accepted as the preferred alternative for treating CML relapse after allo-SCT [23–25]. TKIs can induce complete cytogenetic responses (CCRs) and deep molecular response (MR), even in patients failing DLI, and the treatment is without risk of GVHD [26–28]. Early identification of altered intracellular signaling may be a predictor of response to TKI therapy [29], although whether this is evident also in patients relapsing after allo-SCT remains elusive.

*BCR-ABL1* kinase domain point mutations are detectable in approximately 50% of patients with treatment failure and progression on TKIs, although the incidence of *BCR-ABL1* mutations in patients relapsing after allo-SCT remains uncertain. Although our patient had not been exposed to TKIs previously, we considered this as a relapsed disease. We therefore investigated her for *BCR-ABL1* mutations, but this could not be detected. Hence, treatment with imatinib was initiated. No clinical studies comparing different TKIs in CML relapse after allo-SCT exist, and the choice of TKI should in our opinion be based on risk for potential side effects, patient age, previous exposure to TKI, and potential *BCR-ABL1* mutations.

The use of maintenance TKI treatment after allo-SCT has also been investigated [30–33] and seems to be well-tolerated with a low risk for GVHD [30–33]. TKIs have the potential for changing the kinetics of the disease and should probably be recommended for CML patients allo-grafted [33, 34], also in the advanced phase of the disease [33, 34]. Maintenance therapy may not be required for patients that achieve full donor chimerism and deep MR [25].

To conclude, our case report emphasizes the need for long-time follow-up for allo-transplanted CML patients. Despite stable levels of *BCR-ABL1* transcripts over years, relapse can still occur. Early diagnostics and introduction of TKI therapy are crucial to avoid transformation to more advanced stage of CML.
